# Understanding the function of the GABAergic system and its potential role in rheumatoid arthritis

**DOI:** 10.3389/fimmu.2023.1114350

**Published:** 2023-02-07

**Authors:** Yu Shan, Jianan Zhao, Yixin Zheng, Shicheng Guo, Steven J. Schrodi, Dongyi He

**Affiliations:** ^1^ Department of Rheumatology, Shanghai Guanghua Hospital, Shanghai University of Traditional Chinese Medicine, Shanghai, China; ^2^ Guanghua Clinical Medical College, Shanghai University of Traditional Chinese Medicine, Shanghai, China; ^3^ Institute of Arthritis Research in Integrative Medicine, Shanghai Academy of Traditional Chinese Medicine, Shanghai, China; ^4^ Computation and Informatics in Biology and Medicine, University of Wisconsin-Madison, Madison, WI, United States; ^5^ Department of Medical Genetics, School of Medicine and Public Health, University of Wisconsin-Madison, Madison, WI, United States; ^6^ Arthritis Institute of Integrated Traditional and Western medicine, Shanghai Chinese Medicine Research Institute, Shanghai, China

**Keywords:** rheumatoid arthritis, gamma-aminobutyric acid(GABA)ergic, GABA relatedreceptors, GABA transporter (GAT) systems, inflammation

## Abstract

Rheumatoid arthritis (RA) is a highly disabling chronic autoimmune disease. Multiple factors contribute to the complex pathological process of RA, in which an abnormal autoimmune response, high survival of inflammatory cells, and excessive release of inflammatory factors lead to a severe chronic inflammatory response. Clinical management of RA remains limited; therefore, exploring and discovering new mechanisms of action could enhance clinical benefits for patients with RA. Important bidirectional communication occurs between the brain and immune system in inflammatory diseases such as RA, and circulating immune complexes can cause neuroinflammatory responses in the brain. The gamma-aminobutyric acid (GABA)ergic system is a part of the nervous system that primarily comprises GABA, GABA-related receptors, and GABA transporter (GAT) systems. GABA is an inhibitory neurotransmitter that binds to GABA receptors in the presence of GATs to exert a variety of pathophysiological regulatory effects, with its predominant role being neural signaling. Nonetheless, the GABAergic system may also have immunomodulatory effects. GABA/GABA-A receptors may inhibit the progression of inflammation in RA and GATs may promote inflammation. GABA-B receptors may also act as susceptibility genes for RA, regulating the inflammatory response of RA *via* immune cells. Furthermore, the GABAergic system may modulate the abnormal pain response in RA patients. We also summarized the latest clinical applications of the GABAergic system and provided an outlook on its clinical application in RA. However, direct studies on the GABAergic system and RA are still lacking; therefore, we hope to provide potential therapeutic options and a theoretical basis for RA treatment by summarizing any potential associations.

## Introduction

Rheumatoid arthritis (RA) is an autoimmune-mediated chronic inflammatory joint disease characterized by pain and swelling in the joints of the hands and feet (primarily in the toe), proximal interphalangeal joints, and wrists. Unlike the “hard” swelling of osteoarthritis, this swelling is usually “soft”, and is attributed to synovitis and fluid accumulation (1, 2). The global incidence of RA is approximately 0.24% (3), with a positive correlation with age. Furthermore, RA is a chronic, long-term joint disorder that may persist for decades and even for life (4, 5); however, the pathogenesis of RA remains unclear. Nonetheless, it is understood that in RA, immune tolerance to autologous proteins (such as collagen, wave proteins, and fibrinogen) is disrupted for various reasons, resulting in the formation of autoantibodies against autoantigens, such as anti-citrullinated peptide antibodies, anti-immunoglobulin G antibodies, and autoantigens that cross-react with bacterial or viral antigens (6, 7). It occurs when the immune system mistakenly attacks the tissues in the joints, leading to inflammation, swelling, and destruction of the joint structure (8).

Neovascularization is another hallmark of RA synovitis, in which multiple synovial infiltrates of the joint occur and endothelial cells are activated; furthermore, an expansion of synovial fibroblasts and macrophage-like cells leads to the proliferation of the supra-synovial layer and invasion of the periarticular bone at the cartilage junction, ultimately leading to bone erosion and cartilage degeneration (9). RA is incurable and patients must be treated with disease-modifying anti-rheumatic drugs (DMARDs) to relieve clinical symptoms, improve somatic function, and inhibit the progression of joint damage (10). Commonly used DMARDs include methotrexate, leflunomide, and sulfonamides. Early treatment with combined methotrexate and glucocorticoids followed by other DMARDs with the inhibitors of tumor necrosis factor-α (TNF-α), interleukin-6 (IL-6), or Janus kinase can improve prognosis (11). Treatment aims to reduce disease activity by at least 50% within three months and achieve remission or reduced disease activity within six months, and can prevent RA-related disability with sequential drug therapy if necessary (12, 13). Therefore, understanding and identifying novel targets is important for the clinical management and treatment of RA. There is some evidence to suggest that the GABAergic system may be involved in the development and progression of RA (14). Some studies have found that gamma-aminobutyric acid (GABA) levels are lower in people with RA compared to healthy individuals (15). Additionally, GABA has anti-inflammatory and pain-relieving effects, and some researchers have suggested that abnormal GABA signaling may contribute to the immune system dysregulation that occurs in RA (16).

The GABAergic system is a network of neurons in the brain and central nervous system that produce and use the neurotransmitter GABA (17). GABA is an inhibitory neurotransmitter that helps to regulate the activity of neurons and maintain balance in the nervous system (18). It acts by binding to specific receptors on neurons, causing them to reduce their activity. Some studies have explored the potential role of GABA signaling in RA-related inflammation and pain. For example, some research has suggested that GABA signaling may be involved in the downregulation of T-cell autoimmunity and antigen-presenting cells (APC) activity, which play a role in the development of RA-related inflammation (19). Other studies have found that GABA agonists, which are medications that enhance GABA signaling, may be effective in reducing inflammation and pain in animal models (20). It is important to note that the relationship between the GABAergic system and RA is complex and not fully understood. More research is needed to fully understand the function of the GABAergic system in RA and its potential as a target for treatment. However, the findings of some studies suggest that medications that enhance GABA signaling may be a promising approach for managing RA-related inflammation and pain.

## GABAergic system

The GABAergic system comprises the following components: GABA, glutamate decarboxylase (GAD), GABA-A receptors, GABA-B receptors, and GABA transporters (GATs). GAD is predominantly composed of two isoforms, GAD65 and GAD67. The genes of these isoforms are distributed differently and have multiple functions, and GABA acts by binding to their receptors (17, 21). The balance of the GABAergic system is essential for many physiological aspects, including blood pressure, baroreceptor function, human growth hormone release, weight regulation and feeding, respiratory function, brain function, kidney function, vision, and pancreatic function (22). The most prominent feature of RA is synovial inflammation, which is closely associated with abnormal expression of immune cells and inflammatory factors. Pro-inflammatory cytokines, including tumor necrosis factor (TNF) and IL-6, induce RANKL, prostaglandins, and matrix metalloproteinases, which mediate the pain and swelling of the disease; furthermore, RANKL, TNF, and IL-6 stimulate bone and cartilage (23). Recent studies have shown that immune system cells can produce GABA and express GABA-A ion channels, GATs, and GABA-B receptors (24). The synovium also contains a GABAergic system; specifically, synovial macrophage-like A cells possess a GABA-producing system alongside GAD and GABA-B receptor subunits. Furthermore, the GABAergic system appears to play a functional role in the synovium (14). GABAergic components have been reported to negatively regulate the immune response by affecting the production of pro-inflammatory cytokines and activation of signaling pathways (16, 25). Overall, GABAergic components may offer a new therapeutic approach for inflammatory and autoimmune diseases, including RA. However, the GABAergic system in the synovium has yet to be studied. In this article, we explore the role of GABAergic components in the regulation of inflammation and pain in RA by summarizing the major components of the GABAergic system and their physiological functions, alongside the potential links that exist with RA.

GABA receptors are widely expressed in the central nervous system (CNS) and occupy approximately 20% of the synapses in the cerebral cortex, hippocampus, thalamus, and cerebellum. These are closely linked to the control of cognitive functions such as memory, language, and attention (17); they are also located in the retina, thalamus, hippocampus, pituitary, and gastrointestinal tract, and are associated with visual processing, sleep-wake rhythm regulation, pain perception, memory, learning, hormone regulation, and neuroendocrine gastrointestinal secretion (26). GATs are present in the presynaptic membrane. GAT-1 and GAT-3 are abundantly expressed in the CNS, whereas GAT-2 and betaine/gamma-aminobutyric acid transporter (BGT-1) are expressed in tissues such as the liver, kidney, and intestine (26–28).

### Physiological synthesis of GABA

GABA is the main inhibitory neurotransmitter in the CNS and plays a key role in controlling excitability, plasticity, and network synchronization in the CNS. GABA is present in the CNS alongside many other organs, such as the pancreas, pituitary gland, testes, gastrointestinal tract, ovaries, placenta, uterus, and adrenal medulla (29, 30). GABA synthesis requires GAD, which consists of two isomers (GAD65 and GAD67), encoded by a single gene on chromosomes 10 and 2, respectively, and is the rate-limiting enzyme that catalyzes the conversion of glutamate to the inhibitory neurotransmitter GABA (31). GABA is primarily synthesized *via* two pathways: direct synthesis from glutamic acid catalyzed by GAD65 or GAD67, and synthesis from glutamic acid produced by trichloroacetic acid catalyzed by GAD67. GABA acts through its receptors, primarily GABA-A and GABA-B (32). The GABA-A receptor is an ion-affinity receptor that binds to GABA and opens its complete chloride channel. In contrast, GABA-B receptors are G protein-coupled metabotropic receptors that have a negative effect on presynaptic voltage-activated calcium channels and a positive effect on postsynaptic inwardly rectifying potassium channels (33). GABA signaling in the synaptic cleft can be reuptaken by high-affinity GATs into the presynaptic membrane, thus avoiding GABA overexpression (34).

### GABA receptors

GABA receptors are the major inhibitory neurotransmitters of the vertebrate CNS and are expressed by many neurons in the CNS, as well as other cell types in the periphery, with two main types: GABA-A and GABA-B receptors (35). GABA-A receptors (ionotropic receptors) are ligand-gated ion channels that bind to GABA and open the Cl^-^ channel. GABA-A receptors include many subunit types: α (one-six), β (one-three), γ (one-three), δ, ϵ, θ, π, and ρ (one-three). The most common GABA-A receptor subunit types in the brain are α1, β2, and γ2 (36). GABA-A receptors are recruited to increase acetylcholine release and facilitate transmission; however, GABA-B receptors are activated at high GABA concentrations, thereby decreasing acetylcholine release and transmission (36, 37).

GABA-B receptors (metabotropic receptors) are G-protein coupled receptors that have a negative effect on presynaptic voltage-activated Ca^2+^ channels but a positive effect on postsynaptic inwardly corrected K^+^ channels (38). GABA-B receptors consist of B1 and B2 subunits, and it is generally believed that B1a/B2 heterodimers are localized to presynaptic neurons, while B1b/B2 heterodimers are localized to postsynaptic neurons. GABA-B receptors bind to G protein subsets, which consequently regulate specific ion channels, such as calcium channels, and trigger cyclic adenosine monophosphate cascade responses (39). GABA-B receptors modulate their inhibitory effects by activating the inwardly regulated K^+^ channels, inactivating voltage-stalled Ca^2+^ channels, and inhibiting adenylyl cyclase. Postsynaptic receptors induce slow inhibitory postsynaptic currents that hyperpolarize the membrane and shunt excitatory currents by gating a particular type of K^+^ channel (39, 40).

### GATs

The GAT family, which includes GAT-1, GAT-2, GAT-3, and BGT-1, are important regulators of intracellular and extracellular GABA concentrations; specifically, these transporters mediate the secondary active transport of ion-coupled GABA across the plasma membrane (41). GATs inhibit GABA signaling by translocating GABA to cells and reducing extracellular GABA concentrations, and are potential drug targets in various diseases associated with dysregulated GABA delivery. GAT-1 and GAT-3 are predominantly expressed in the CNS where they regulate GABA activity (42), whereas GAT-2 is mainly located in peripheral organs, especially in the liver and kidney, and is thought to be a GABA and taurine transporter in the liver (43). GAT-1 is a highly conserved molecule encoded by solute carrier family 6 member 1, which transports GABA *via* Na^+^ and Cl^-^. As the major GAT in the brain, in addition to being involved in a wide range of brain functions, GAT-1 has been implicated in the pathophysiology of several neuropsychiatric disorders, including anxiety disorders, depression, epilepsy, Alzheimer’s disease, and schizophrenia (44).

## Potential link between GABAergic system and RA

The GABAergic system may act as a bridge between the nervous system and the immune system. As an inhibitory neurotransmitter in the CNS, the main role of GABA is to reduce the excitability of neurons throughout the nervous system (18). Activation of GABA and other ligands by the GABA receptor induces a conformational change in the receptor that increases axonal K^+^ conductance, thereby accelerating action potential repolarization and leading to transient secondary inhibition of calcium by decreasing the degree of calcium channel activation (45, 46). GABAergic system plays an important role in the function of immune cells mainly being influenced by GABA signaling. Recent studies have highlighted the immune function of GABA, suggesting cross-talk between the nervous and immune systems (47, 48). T cells express many neurotransmitter receptors, and their expression is regulated by T cell receptor activation, cytokines, and neurotransmitters themselves (49). The GABAergic system can negatively regulate immune responses, especially T cell–mediated immune responses, by affecting the production of pro-inflammatory cytokines and the activation of signaling pathways, such as mitogen-activated protein kinase and nuclear factor kappa-light-chain enhancer of activated B cells (NF-κB) pathways (50). These results may indicate that the GABAergic system offers a new therapeutic approach for inflammatory and autoimmune diseases and requires further evaluation, especially regarding RA treatment.

### GABA/GABA-A receptor may inhibit inflammation in RA

Central and peripheral GABA-A receptors play a major role in inflammation; specifically, GABA inhibition reverses the pathological pain state in mice, and GABA-A reduces the production of pro-inflammatory mediators, thereby ameliorating the symptoms of inflammation (51). GABA/GABA-A receptors may induce RA inflammation by affecting T cell and macrophage populations. Mechanistically, GABA receptors firstly interact with KCNN4 to induce Ca^2+^ entry, resulting in activation of nuclear factor κB signal, and finally promotes macrophage invasion by inducing CXCL5 and CCL20 expression (52). This functional GABAergic system acts as a regulator of T cell activation (16). GABA is not only an inhibitory neurotransmitter but also an immunomodulator. GABA binding to GABA-A receptors directly affects the function of antigen-presenting cells and inhibits the production of inflammatory cytokines by T cells (53). Binding of GABA to GABA-A receptors also inhibits the proliferation of antigen-specific T cells and suppresses the production of IL-6, IL-12, inducible nitric oxide synthase, IL-1β, and TNF-α (52, 54). Furthermore, activation of GABA-A receptors inhibits the release of TNF-α and IL-6 from alveolar macrophages induced by lipopolysaccharide (55).

P38/MAPK may be a key player in the link between the GABA/GABA-A axis and inflammation in RA. P38 is a tyrosine phosphoprotein kinase isolated and purified from mammalian cells stimulated by endotoxin; this enzyme is the most important member of the mitogen-activated protein kinase (MAPK) family in terms of inflammatory response mediation (56). Key enzymes of the P38/MAPK pathway include mitogen-activated protein kinase kinase (MKK)-3, MKK6, and transforming growth factor-β activating kinase (TAK) (57). TAK activates MKK4, which in turn activates P38/MAPK that phosphorylates and activates many protein kinases and transcription factors (58). P38/MAPK activates signal transducer-activator of transcription 4 (STAT4) and NF-κB and promotes the release of inflammatory factors such as TNF-α, IL-1β, and IL-6 (59). Inflammatory stimuli, such as TNF-α, platelet-activating factor, and interleukins, induce P38 activation in endogenous immune cells such as monocytes, endothelial cells, and neutrophils (60, 61). When synovial disruption begins, somatic afferent pain signals received by the spinal cord could lead to stress-induced kinase release; these pain and cytokine signals activate P38/MAPK, which induces the upregulation and release of pro-inflammatory cytokines such as IL-1, IL-6, and matrix metallopeptidase 3 (MMP3) into the periphery (62, 63). A relationship exists between the CNS and peripheral immune response; specifically, afferent pain signals help the CNS propagate the inflammatory response, which may influence the development of peripheral arthritis (64). GABA is a major inhibitory neurotransmitter in the CNS that downregulates P38 activity to reduce the production of proinflammatory cytokines. For example, GABA prevents IL-6 release by inhibiting P38/MAPK in glioma cells, potentially affecting the inflammatory response in RA (65, 66).

### The GABAergic system may be involved in regulating the pain response in RA

The GABAergic system has been suggested to be involved in the pain response in RA. Pain is one of the most important manifestations of RA, and is thought to be caused by joint inflammation (67). Inflammatory pain is predominantly due to local joint synovial inflammation caused by pro-inflammatory cytokines. This inflammation leads to activation of afferent nociceptive fibers and transmission of “pain” signals to the dorsal horn of the spinal cord, *via* the spinothalamic tract, to the thalamus (68, 69). However, clinical studies have shown that even when inflammation is controlled, patients with RA may continue to experience non-inflammatory pain (70). The main factors causing non-inflammatory pain are yet to be determined. Nonetheless, they are understood to be related to structural changes in the patient’s joint environment, continued disease progression, ectopic secretions, and peripheral nerve damage and dysfunction due to increased excitability of damaged afferent injury receptors, which manifest as neuropathic pain (71, 72). GABA is an inhibitory neurotransmitter; therefore, selective loss of GABAergic interneurons after peripheral nerve injury is thought to be the underlying cause of inhibitory signal loss in RA. Furthermore, reduced inhibitory neurotransmission is a key feature of chronic pain states (73). There are two possible causes for this decrease in inhibitory neurotransmission: a decrease in GABA and its synthase caused by apoptosis of GABAergic neurons in the spinal cord (74, 75) and depletion of GABA in the synaptic gap (76). GABA-A and -B receptors and GAT proteins appear to be involved in the pathophysiology of the chronic pain associated with RA (73). Currently, several studies have investigated the positive modulators of these two receptors and used them for various types of pain; furthermore, a successful testing phase in animal models has recently been achieved (77). GABA-A and glycine receptors are key elements of the spine that control injury perception and pain. Impaired function of these receptors may contribute to the development of chronic pain; therefore, restoring their normal function through aggressive modulators has become an effective treatment for chronic pain syndromes (78). In the context of RA, the pain response in patients with RA may be modulated through the GABAergic system. Therefore, some small-molecule inhibitors or activators currently targeting the GABAergic system may be potentially beneficial in RA.

### GABA-B receptors may be associated with inflammation in RA

Neutrophil is one of the key inflammatory effector cells of RA.GABA-B receptors are expressed in neutrophils and play an important role as chemotactic receptors in the inflammatory response (79). GABA-B receptors are also metabotropic receptors. Unlike ionotropic GABA-A receptors, which utilize rapid synaptic transmission, GABA-B receptors are heterodimers consisting of subunits encoded by *GABBR1* and *GABBR2* (80). The coding region of GABA type B receptor subunit *(GABBR)-1* is located on chromosome 6, 6p21.3, and the major histocompatibility complex (MHC) located in this region is associated with multiple sclerosis, Alzheimer’s disease, schizophrenia, narcolepsy, epilepsy, and RA (81). The specific MHC, class II, DR beta 1 alleles are strongly associated with susceptibility to RA; this susceptibility is likely due to their role in presenting arthritogenic polypeptides. However, the linkage disequilibrium of the *GABBR1* gene polymorphism with these alleles is not as expected, as the distance between the two motifs is three Mb (82). Thus, the observed genetic association with this region suggests that *GABBR1* plays an independent role in genetic susceptibility to RA (83).

In yeast glycan-induced arthritis, GABA-B receptors are involved in neutrophil migration to the knee, similar to GABA; this migration may be associated with P38/MAPK activation in the spinal cord (84). Spinal cord inhibitory signaling may downregulate P38/MAPK and reduce pro-inflammatory cytokine production. Furthermore, any changes that affect this negative regulation, such as SNP alleles or haplotypes in *GABBR1*, may allow P38/MAPK to continue and worsen RA pathology in an uncontrolled manner (82). Although *GABBR1* polymorphisms have not been experimentally characterized in RA patients, computational analysis suggests that *GABBR1* encoding multiple isoforms, mutations, and several genes that potentially affect selectively spliced protein structures may be associated with RA progression (85).

### GATs promote inflammation

Members of the GAT family, including GAT-1, GAT-2, GAT-3, and BGT-1, may contribute to inflammation. Multiple inflammatory factors, such as IL-6, IL-1β, and TNF-α are present in RA (86). The expression of GAT-1 and GAT-3 is closely associated with inflammatory factors, such as IL-6, IL-1β, and TNF-α, and may show a positive correlation with inflammation (87). IL-1β and TNF-α were found to upregulate GAT-1 and GAT-3 expression through the MAPK pathway, which subsequently increased IL-6 levels and further upregulated GAT-1 and GAT-3 expression; alternatively, inhibition of IL-1β and TNF-α receptors attenuated this GAT-1 and GAT-3 expression (88). GATs may be associated with subpopulations of lymphoid T cells and have been determined to regulate cytokine production and T cell proliferation. The gene transcripts of two cotransporters, GAT-1 and GAT-2, have been detected in immune cells and identified in human peripheral blood lymphocytes (89). GABA inhibits Th1 cell–mediated DTH responses *in vivo* and participates in T cell immunity *via* the GAT and GABA receptors. For example, GAT-1 is expressed only on antigen-activated T cells and downregulates the proliferation and expression of CD4^+^ T cells (90). These findings suggest that GAT-1 is a key regulator of the T cell–mediated immune response. Additional evidence suggests that T cells expressing GAT-2 and GAT-2 deficiency promote T helper 17 cell (Th17) responses through the activation of GABA-mammalian target of rapamycin signaling; specifically, in a mouse model of infection, GAT-2 deficiency was observed to enhance the differentiation of naive T cells into Th1 cells (91).

GAT-2 is primarily pro-inflammatory in RA. Furthermore, Interferon (IFN)-γ is an important effector of RA (92) that induces GAT-2 expression in macrophages (93). A systematic evaluation and meta-analysis of common trace metals in RA found that serum copper levels were elevated in RA and higher in patients with active RA. These levels were positively correlated with erythrocyte sedimentation rate and morning stiffness, and were negatively correlated with hemoglobin levels and considered to be adjunctive markers for disease assessment (94, 95). Therefore, GAT-2 may promote inflammatory responses by being associated with abnormally high copper levels in RA. GAT-2 deficiency attenuates macrophage-mediated inflammatory responses *in vivo*, including lipopolysaccharide-induced sepsis, infection-induced pneumonia, and high-fat diet-induced obesity (25, 96). GAT-2 deficiency also decreases IL-1β production in pro-inflammatory macrophages. This mechanism may involve enhancement of the betaine/S-adenosylmethionine/hypoxanthine metabolic pathway by increasing DNA methylation in its promoter region to inhibit the expression of the transcription factor zinc finger protein 354C (*ZNF354C).* This zinc finger protein suppresses inflammasome formation and inhibits M1 macrophage polarization by targeting the expression of oxidative phosphorus-related genes (93). According to previous reports, activation of the GABAergic system in macrophages enhances autophagy activation, phagosome maturation, and antimicrobial response to *Mycobacterium* infection (97). Macrophage autophagy in the context of RA is an important mechanism that may co-mediate the abnormal immune response of macrophages with the GABAergic system (98).

## Future perspectives and challenges: Clinical trials related to GABAergic system components

Relevant clinical trials of the major components of the GABAergic system mainly involve GABA, GABA receptor agonists and antagonists, and GABA agonists and antagonists (**Table 1**). Although there have been no direct clinical studies in the context of RA, ongoing clinical trials are currently providing information regarding the development of clinical small-molecule GABA drugs for RA. First, GABA and GABA-A receptor agonists can inhibit the immune response of immune cells to stimuli, which may be important for remission of inflammation (99). For example, honokiol (a GABA-A receptor modulator) relieves inflammatory arthritis and allergic asthma by affecting cytokine expression of cytokines (100). Additionally, a series of immunological abnormalities have been reported in T1DM patients, involving production of autoantibodies, glutamic acid decarboxylase (GAD-65), tyrosine phosphatase-associated islet antigen 2, zinc transporter protein 8, and insulin; furthermore, there is an altered ability of regulatory T cells (Treg cells) to inhibit the action of effector T cells, which play a key role in the immune destruction process (101). GABA also acts on GABA-A receptors in pancreatic alpha cells, thereby inhibiting glucagon secretion, suppressing inflammation, and increasing the number of regulatory T cells. In RA, Treg cells are important for the proliferation of inflammatory cells (Th17) and suppression of inflammatory factor secretion. Therefore, GABA has potential therapeutic implications for RA by increasing the number of regulatory T cells. Furthermore, the application of activators or inhibitors of the GABAergic system may have potential therapeutic effects on the pain response in RA. For example, studies based on animal models of inflammatory or neuropathic pain have found that selective positive modulators of α2 and α3 subtypes of GABA-A receptors may reverse the loss of postsynaptic GABA-A receptor-mediated spinal cord inhibition, leading to analgesia (102). GABA-B receptors are highly expressed in the structure of the pain pathway, suggesting their involvement in different levels of pain signaling; thus, they have long been considered valuable targets for the treatment of chronic pain (103). Activation of GABA-B receptors leads to hyperpolarization and reduced firing frequency, increased mediation of inhibitory neurotransmission, and production of analgesia in the brain and spinal cord (104). The GABA-B receptor agonist, baclofen, is widely used in clinical practice for the treatment of chronic pain. In the presence of reduced endogenous GABAergic tone, the GABA-B receptor agonist baclofen exerts an anti-injury sensory effect and releases GABA from the cortical precursors of GABAergic interneurons transplanted into the medial ganglion bulge, ultimately reversing neuropathic abnormal pain (105). GAT proteins are highly dynamic in different cell types and are responsible for the recycling and reuptake of GABA, thereby terminating inhibitory signaling (106). Studies have reported that inhibition of GAT reuptake enhances GABAergic neurotransmission and has an inhibitory effect on the release of aspartate and glutamate in the dorsal spinal cord, thereby inhibiting the release of pro-nociceptive neurotransmitters to achieve analgesia (107).

**Table 1:** Clinical trials of GABAergic system components.

Name	Condition or disease	ClinicalTrials.gov number	Sponsor	Interventions	Primary outcomes	Phase
GABA	Pain Healthy subjects	NCT02928328 NCT04086108	Aalborg University Wageningen University	GABA/lorazepam GABA/Tomato	Pain Intensity Rating Area under the plasma concentration versus time curve (AUC) of plasma-time curves of GABA	Not Applicable Not Applicable
	Diabetes Mellitus, Type 1 Diabetes Mellitus, Type 1 Healthy subjects Diabetes Mellitus, Type 1 Sleep Disturbance Type I Diabetes Epilepsy	NCT04375020 NCT03635437 NCT04303468 NCT03721991 NCT04857021 NCT02002130 NCT04144439	Ministry of Health and Population, Egypt Uppsala University Hospital Wageningen University Steno Diabetes Center Copenhagen Amorepacific Corporation University of Alabama at Birmingham Tanta University	GABA GABA/Alprazolam GABA/Placebo GABA/Placebo GABA/Placebo GABA/Placebo/GAD-alum GABA	GABA decrease anti gad antibodies GABA improve c peptide levels Adverse events possibly or probably related to GABA treatment Postprandial glycemic response during a 2-hour oral glucose tolerance test (OGTT) Insulin production, c peptide production during meal stimulation Change of total sleep time of polysomnography. Change of sleep latency of polysomnography. Change of NonREM stage 3 of polysomnography Compare the effect of oral GABA or oral GABA/GAD combination administration on pancreatic beta cell function by quantitative C-peptide secretion Number of seizures [Time Frame: 6 months]	Not Provided Phase 1 Not Applicable Not Applicable Not Applicable Phase 1 Phase 4
AZD7325	Healthy subjects	NCT02135198	University College, London	AZD7325/Placebo AZD7325/Placebo AZD7325/Placebo AZD7325/Placebo Baclofen/Placebo Baclofen Baclofen/Placebo Arbaclofen/Placebo Clobazam/Clonazepam/Tolterodine	Change in conventional measure of percentage short interval intracortical inhibition (SICI) at an interstimulus interval (ISI) of 2.5 ms and conditioning stimulus intensity of 70 percent of resting motor threshold	Phase 1 Phase 1 Phase 2 Not Applicable Not Applicable Phase 2 Phase 1 Not Applicable Phase 3
	Healthy subjects Autism Spectrum Disorder Autism Spectrum Disorder	NCT02530580 NCT01966679 NCT03678129	University College, London University of California, Los Angeles King’s College London Imperial College London National Institute on Drug Abuse Assistance Publi que-Hôpitaux de Paris King’s College London University Hospital Inselspital, Berne		Change in peak grip force in an object manipulation task [Time Frame: from baseline at 1, 2, and 3 hours after the study medication] Electroencephalogram [Time Frame: week 6] Neurochemical response to GABAergic stimulation.	
Baclofen Arbaclofen Clobazam	Healthy subjects Cocaine-Related Disorders CHEYNE Stokes Respiration Autism Spectrum Disorder Pain	NCT01563224 NCT00218166 NCT01095679 NCT03594552 NCT01011036			EEG spectral power in theta band [Time Frame: Change from baseline to 4 hours after dosing] Progressive-ratio break point [Time Frame: Measured during each experimental session] Decrease in the coefficient of variation of the period of the ventilatory cycle Neurochemical response to GABAergic stimulation Area of hyperalgesia on the forearm	
Flumazenil Iomazenil	Fragile X Syndrome or Idiopathic Intellectual Developmental Disorder Primary/Idiopathic Hypersomnia Cognitive Dysfunction	NCT04308954 NCT01183312 NCT00611572	Stanford University Emory University Yale University	Flumazenil Flumazenil Iomazenil	Non-displaceable binding potential of F18 FMZ GABA (A) receptor density in fragile X syndrome (FXS) patients relative to control group comprising individuals with IDD Change in Psychomotor Vigilance Task (PVT) Median Reaction Time P300 as an ERP measure Mismatch Negativity	Phase 1 Phase 2 Phase 1

## Discussion

**Figure 1:**
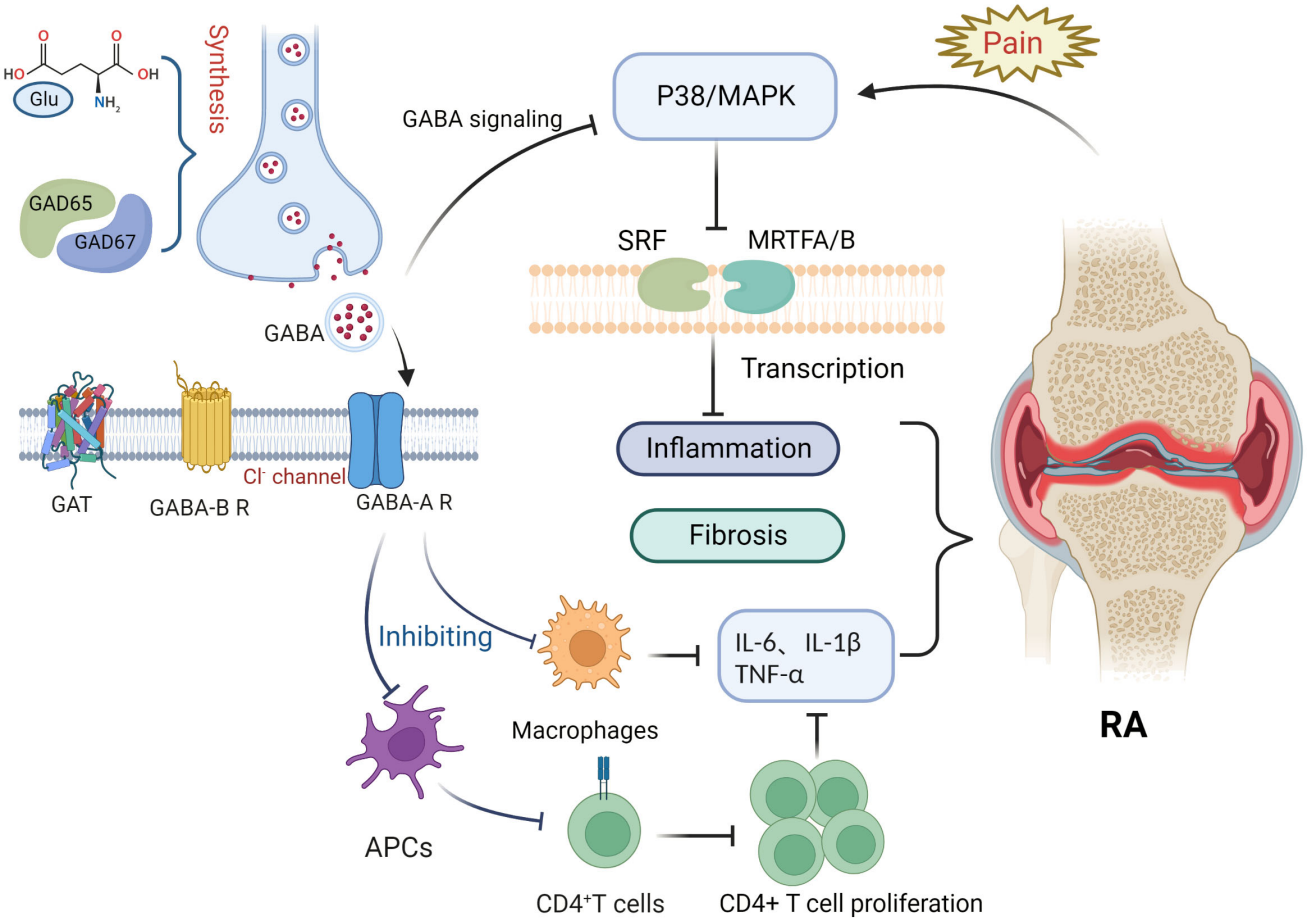
Potential role of the gamma-aminobutyric acid (GABA)ergic system in rheumatoid arthritis. Glutamate interacts with glutamate decarboxylase (GAD65 and GAD67) to produce gamma-aminobutyric acid (GABA). Binding of GABA to GABA-A receptors inhibits macrophage activation and decreases the release of inflammatory factors such as IL-6, IL-1β, and TNF-α. Antigen presentation by antigen-presenting cells, however, is impaired, inhibiting CD4^+^ T cell proliferation and differentiation and reducing the expression of inflammatory factors such as IL-6, IL-1β, and TNF-α. Pain signaling activates the P38/MAPK pathway, whereas GABA binding to GABA-A receptors inhibits P38/MAPK. The P38/MAPK signaling pathway contributes to inflammation and is involved in the activation of myocardin-related transcription factor A (MRTFA), myocardin-related transcription factor B (MRTFB), and serum response factor (SRF) that played key roles in fibroblast activation.

This article focuses on the physiological role of the GABAergic system, its components, and the possible mechanisms of its influence in RA pathology (**Figure 1**). RA is an inflammatory disease that manifests in peripheral joints and connects the nervous system to the immune system *via* spinal P38/MAPK. GABA is not only a neurotransmitter, but also an immunomodulator that downregulates P38/MAPK activity to reduce the production of pro-inflammatory cytokines in RA joints, inhibit pro-inflammatory T cell value, and increase the number of regulatory T cells. Through MAPK phosphorylation, the GABA-A receptor inhibits the production of inflammatory cytokines *via* T cells and suppresses the proliferation of effector T cells, thereby inhibiting the inflammatory progression of RA. In contrast, the GABA-B receptor may be relevant to the pathogenesis of RA due to its unique coding region. GAT expression is positively correlated with inflammatory factors, with an increase in GATs promoting high expression of inflammatory factors and vice versa. GAT-2 deficiency enhances the differentiation of naive T cells into Th1 cells. The GABAergic system also plays an important role in analgesia, as GABA is an inhibitory neurotransmitter that negatively regulates pain signaling; similarly, its derivative, gabapentin, is also used in the treatment of pain. Both GABA receptors and its modulators are potential targets for pain treatment; consequently, studies surrounding the use of GAT inhibitors for pain treatment are ongoing. Therefore, we can see that the GABAergic system plays an important role in the pathogenesis and disease progression of RA, and in addition, GABA has excellent prospects for application in the control of RA-related pain. Nonetheless, there is still no relevant research about the direct link between GABA and the pathogenesis of RA. Further exploration regarding other aspects of the research, such as the nervous and immune systems, could improve our understanding of the overproduction of cytokines, underlying genetic information, and related signaling pathways in RA or neurodegeneration, which will ultimately provide a multidisciplinary understanding of RA and neurological diseases.

## Author contributions

YS and JZ are responsible for the collection, collation, and writing of the original manuscript. YZ is responsible for the collection. SG, SS, and DH are responsible for the concept development, revision, and manuscript review. All authors contributed to the article and approved the submitted version.
